# What is a human embryo? A new piece in the bioethics puzzle

**DOI:** 10.3325/cmj.2014.55.669

**Published:** 2014-12

**Authors:** Ińigo de Miguel Beriain

**Affiliations:** Interuniversity Chair in Law and the Human Genome, University of Deusto, University of the Basque Country, UPV/EHU, Bilbao, Spain

## The embryo as the subject of bio-objectification process

The controversy surrounding the human embryo is a long-standing one. Thirty years ago, there was already an intense debate over moral and legal status of the human embryo and its moral, legal, and political implications. This controversy is still very much alive. In fact, the emergence of modern technologies in the recent years has made the debate even more complicated. Today, we not only debate how to treat the human embryo, but we are also faced with a renewed discussion about what the embryo is. The issue cannot be addressed from a purely scientific point of view since it includes elements that go far beyond biology. Moreover, the embryo has undergone a process of bio-objectification, a process “wherein life-forms or living entities are first made into objects, become possible, through scientific labor and its associated technologies, and then come to be attributed with specific identities” (1). The embryo has become an extremely polemic entity, defying the traditional boundaries as well as the traditional scientific, legal, and moral paradigms. All these paradigms were created as a consequence of the definition of the embryo, and subsequently have been based on the doubtful assumption that embryo is clearly defined. In reality we have to deal with a situation where “embryo” has different meanings, depending on the geographical scope, ideological framework, and type of social science addressing the issue.

## The changes to the concept of the embryo introduced by new biotechnologies

Understanding the process of human embryo bio-objectification will surely take some time. Back in the early 1990s, the definition of the human embryo was based on a purely biological fact: the mixture of the male and female gametes, that is, what science has traditionally called fertilization. The entity arising as a result of human fertilization was considered an embryo. In some cases, taglines such as “normal fertilization” or “successful fertilization” were added to the description so as to avoid, for example, the belief that hydatiform moles were embryos. However, beyond these small disagreements, this was considered a satisfactory way of defining the human embryo.

The scenario, however, changed dramatically in February 1997, when the *Nature* published an article on the birth of Dolly (2), the first cloned mammal in history. It became obvious that mammals can be generated by nuclear transfer techniques, a possibility that in the future can be applied to humans, as the recent achievements by Mitalipov’s team have suggested (3). At that point the traditional definition of human embryo suddenly became obsolete. Since it was possible to create human beings through procedures different from fertilization, the traditional definition led to absurd results. For instance, we would have to consider a cloned being a rare, biological exception, if it were in fact a being in spite of its never having been at the stage of an embryo (as cloning involves no fertilization).

Under these circumstances, many countries began to change their legal definition of the embryo to adapt it to the new possibilities. The Netherlands (4), Belgium (5), and Germany (6), among others, based the definition of the embryo on the idea of potentiality, considering the cell or group of cells to be capable of generating a human being. On the other hand, some countries did not change their definition. For instance, in the case of the United Kingdom, the scarce legal protection offered to the embryo made modifications quite unnecessary. A major change of the legal definition of the embryo could have raised relevant issues on the status of cybrids (human/animal embryos), which were considered embryos (and consequently used for research purposes) under the UK legislation. Changing the definition would possibly paralyze their use due to legal vagueness.

Spain and Finland, among others, also opted to maintain the traditional definition of the embryo for political reasons, somewhat different from those in of the UK (7). In 2007, both countries had already ratified the Convention for the Protection of Human Rights and Dignity of the Human Being with regard to the Application of Biology and Medicine (otherwise known as the Convention on Human Rights and Biomedicine or the Oviedo Convention), whose Article 18.2 prohibits the creation of human embryos for research purposes. However, none of them (especially Spain) wanted to block the research related to stem cells’ nuclear transferability. Hence, they simply decided (explicitly in the case of Spain, implicitly in the case of Finland) to state that the product of a nuclear transfer would never be an embryo because the procedure did not include any kind of fertilization. This approach represents an extremely ingenious solution though clearly a sophism. For this purpose, Finland preferred not to alter the existing law on biomedical research. However, Spain’s Law 14/2007, of 3 July 2007 on Biomedical Research (Article 3, Letter l) includes a definition of the embryo that directly referred to fertilization: an embryo is *“*a phase of embryonic development from the moment in which the fertilised oocyte is found in the uterus of a woman until the beginning of organogenesis and which ends 56 days from the moment of fertilisation, with the exception of the computation of those days in which the development could have been stopped*.”*

However, the “Spanish approach” has not been adopted by any other country. Moreover, the tendency to define the embryo based on the idea of potentiality has been embraced in some way by a major judgment of the Court of Justice of the European Union in the case of Oliver Brüstle vs Greenpeace (8). Here, this court stated that:

“Any human ovum after fertilisation, any non-fertilised human ovum into which the cell nucleus from a mature human cell has been transplanted and any non-fertilised human ovum whose division and further development have been stimulated by parthenogenesis constitute a ‘human embryo’ within the meaning of Article 6 (2)(c) of the Directive” (point number 38 of the judgement).

The change was due to a single reason:

“Although those organisms have not, strictly speaking, been the object of fertilisation, due to the effect of the technique used to obtain them they are, as is apparent from the written observations presented to the Court, capable of commencing the process of development of a human being just as an embryo created by fertilisation of an ovum can do so.” (point number 36 of the judgement)

Nevertheless, the court decision did not end the debate. Instead, it intensified it, as it introduced a new factor, which challenged both the traditional definition of embryo and the new approaches included in the German and Belgian laws. It differed from the traditional definition as it accepted the idea that an embryo could be created not only by fertilization, but also through biotechnological techniques. It differed from other proposed definitions as it did not use the idea of the cell line’s potentiality as the key factor, but directly assumed that all cell lines produced by fertilization, by nuclear transfer, and even by parthenogenesis, must be considered human embryos.

The definition of the human embryo continues to be a major issue in the currently existing legal frameworks. Currently, the traditional definition coexists with the new one based on the idea of potentiality, which has been partially – but only partially – reflected in the jurisprudence of the European Union’s Court of Justice. Technological developments affect traditional legal frameworks and oblige us to arrive at new levels of consensus.

## The concept of the embryo in the ethical framework

In the ethical arena, the situation regarding the definition of the embryo appears quite similar. Most authors consider the traditional definition as a “misleading anachronism” (9). However, this does not imply that they are looking forward to embracing the proposed alternatives. The Court of Justice’s proposal can hardly be supported, as in the same category it includes entities different in their characteristics and potentialities. Indeed, it is difficult to understand why we should consider that a cell produced by a successful fertilization or transfer is the same as a cell that could never develop into a more complex entity. But this is exactly what the Court has done.

Definitions based on the idea of potentiality have created new controversies for various reasons. First, the idea of potentiality inherently involves the necessity to determine a final point that the entity must reach. In the case of the legal framework, that point is birth, which makes sense when we keep in mind that laws have traditionally attached considerable importance to this fact. However, the relevance of this concrete moment remains dubious from an ethical point of view. Leaving aside the unconvincing theories of social recognition, as defended by Jürgen Habermas (10), among others, almost nobody would support the legal approach. From the point of view of biology, there are no relevant differences in a child before and after birth. Moreover, a definition of the embryo based on its potentiality to reach the moment of birth creates questions that are extremely uncomfortable. For instance, can an embryo be an entity resulting from fertilization that cannot become a born offspring only because it suffers from a pathology that impedes it? In such cases, are we facing a non-embryo or a sick embryo? What if we could cure the pathology? Would this create an embryo or simply cure it?

These types of issues clearly show the difficulty of accepting, from an ethical point of view, the conceptual turn included in some legal frameworks. The existence of these issues also explains why some authors are currently trying to offer new approaches to this issue. The most notorious attempt in this direction is probably the DIANA criteria developed by Suarez (11). They are based on the idea that the proper biological potential for developing neural activity specific to human body’s spontaneous movements provides the observable basis for ascertaining the presence of a spiritual soul. Thus, only the presence of DIANA insufficiencies in a cellular entity’s genomic information (insufficiencies that Directly Inhibit the Appearance of Neural Activity) ought to be considered a sure sign that such a cellular entity is not spiritually ensouled and, therefore, not a human being. In any other case, we should consider a group of cells originated by any of the ways that might create a human embryo is, in fact, a human embryo. However, this attempt is clearly far from being accepted by the bioethicist community.

## Conclusion

The discussion on the status of the human embryo has a long history. We are not likely arriving near to any kind of definitive conclusions. Instead, the increasing complexity of the scientific scenario is provoking the opposite. Thus, it seems undeniable that bio-objectification of human embryo increases as the new technologies become available. New scenarios could appear wherein most of our shared beliefs and even our most consolidated scientific evidence might be challenged. The tensions regarding the concept of embryo are only a good example of what is yet to come.
